# Development of Poly(sorbitol adipate)-*g*-poly(ethylene glycol) Mono Methyl Ether-Based Hydrogel Matrices for Model Drug Release

**DOI:** 10.3390/gels10010017

**Published:** 2023-12-23

**Authors:** Haroon Rashid, Henrike Lucas, Karsten Busse, Jörg Kressler, Karsten Mäder, Marie-Luise Trutschel

**Affiliations:** 1 Institute of Pharmacy, Martin Luther University Halle-Wittenberg, D-06120 Halle (Saale), Germany; 2 Department of Chemistry, Martin Luther University Halle-Wittenberg, D-06120 Halle (Saale), Germany

**Keywords:** poly(sorbitol adipate), PSA-*g*-mPEG, PEG, enzymatic polymerization, Steglich esterification, hydrogels, polymer networks, swelling, solute release, drug delivery

## Abstract

Hydrogels were prepared by Steglich esterification and by crosslinking pre-synthesized poly(sorbitol adipate)-*graft*-poly(ethylene glycol) mono methyl ether (PSA-*g*-mPEG) using different-chain-length-based disuccinyl PEG. PSA and PSA-*g*-mPEG were investigated for polymer degradation as a function of time at different temperatures. PSA-*g*-mPEG hydrogels were then evaluated for their most crucial properties of swelling that rendered them suitable for many pharmaceutical and biomedical applications. Hydrogels were also examined for their Sol-Gel content in order to investigate the degree of cross-linking. Physical structural parameters of the hydrogels were theoretically estimated using the modified Flory–Rehner theory to obtain approximate values of polymer volume fraction, the molecular weight between two crosslinks, and the mesh size of the hydrogels. X-ray diffraction was conducted to detect the presence or absence of crystalline regions in the hydrogels. PSA-*g*-mPEG hydrogels were then extensively examined for higher and lower molecular weight solute release through analysis by fluorescence spectroscopy. Finally, the cytotoxicity of the hydrogels was also investigated using a resazurin reduction assay. Experimental results show that PSA-*g*-mPEG provides an option as a biocompatible polymer to be used for pharmaceutical applications.

## 1. Introduction

In the last few decades, biodegradable polymers have been proven crucial in the significant development and advancement of various drug delivery systems [1,2]. Controlled drug delivery, vaccines, nucleic acids, proteins, anticancer medications, tissue engineering, and regenerative medicine are areas where recent advancements have been made [3,4,5,6,7,8]. Biodegradable polymers act as temporal materials that break down into less complex elements, removing the dangers of long-term foreign material presence. Through enzymatic or non-enzymatic mechanisms, they break down into biocompatible and harmless byproducts [9]. Biodegradable natural polymers have been used in medicine for a long time. Investigations into synthetic biodegradable polymers date back to the 1960s and 1970s, when polyesters like poly(glycolic acid), poly(D,L-lactic acid) (PLA), and poly(D,L-lactic-co-glycolic acid) (PLGA) were developed for use as biodegradable materials for various biomedical purposes [6,10]. Since then, PLA and PLGA have been used for various drug delivery purposes; however, researchers also report various shortcomings related to PLA and PLGA. These include complex release and degradation patterns [11,12,13], self-catalyzed polymer degradation [13,14], and the development of microenvironments with an acidic nature. According to research, extremely low pH readings, frequently less than 2, have been recorded under in vivo and in vitro conditions [15,16,17]. Another disadvantage that these aliphatic polyesters have is their lack of free functional groups that can be used to link with various polymers or drugs [18,19,20]. This lack confines them to be modulated for enhanced or desired material characteristics [21].

Enzymatic polymerization provides us with one of the alternatives to the above-mentioned polymers that utilizes green chemistry to synthesize functional polyesters in which enzymes are being used as biocatalysts [22,23,24,25,26]. The enzymes enable us to produce aliphatic polyesters without exposure to metal-based catalysts that carry toxicity risk in a conventional polymerization process [22]. Additionally, even when several monomers bearing OH groups (more than two) are utilized, enzymes enable the selective synthesis of linear polymers with minimal branching [24,27]. Therefore, enzymatic polymerization can enable us to produce polyesters with multiple free functionalities. This gives us the advantage of overcoming the usage of standard protection/deprotection chemistry, which is generally used for the conventional synthesis of functional aliphatic polyester [28,29].

The Kressler group, Maeder group, and a few other groups have been working on enzymatically polymerized aliphatic polyester, poly(glycerol adipate) (PGA), to develop numerous drug delivery systems, such as nanoparticles [30,31,32], micelles [33], and microparticles [34] and as polymer-drug conjugates [17]. Steiner et al. developed a microparticulate drug delivery system using poly(glycerol adipate) as the base, and by grafting it with acyl side chains. The system was employed for releasing a model drug, dibenzoyl thiamine, and a sustained drug delivery profile was observed. This drug release pattern was attributed primarily to the modification involving fatty acids that is enabled by the single pendant functionality of poly(glycerol adipate) [34]. Wersig et al. prepared poly(glycerol adipate)-based nanoparticles by utilizing its free functionality and conjugating it with the drug to achieve the controlled delivery of an anti-inflammatory drug, indomethacin [17].

Over the years, hydrogels have also proved to be a versatile drug delivery system, mainly because of their strong affinity towards water and their elastic nature that mimics natural tissues. This property of hydrogels to attract water largely depends on the chemical functional groups that are present in the polymer backbones, e.g., hydroxyl groups, carboxyl groups, amide groups, etc. [35,36]. Over the course of research, hydrogels utilizing PLA [37], PLGA [38], and poly(glycerol sebacate) (PGS) [39] have been formulated to serve a wide array of pharmaceutical and biomedical purposes. Unfortunately, these polymers are encumbered by the limitations outlined earlier. Furthermore, enzymatically synthesized poly(glycerol adipate), used previously in drug delivery systems, comprises a single pendant hydroxyl group in each of its monomeric units, imparting an amphiphilic nature [19] but lacking water solubility [40].

Ongoing research has been aimed at synthesizing poly(sorbitol adipate) (PSA), which distinguishes itself from poly(glycerol adipate) by incorporating four pendant functionalities within each monomeric unit, rendering it water-soluble in character and providing more choice to modulate the material characteristics. More importantly, this work is pioneering the use of enzymatically synthesized aliphatic polyesters to fabricate crosslinked polymeric hydrogels. Utilizing the multiple pendant functionalities of PSA, it has been copolymerized with PEG to enhance its swelling and diffusion properties, and later crosslinked to form hydrogels via Steglich esterification [24]. This work is also aiming here to investigate PSA-based hydrogels through the equilibrium swelling theory by Flory–Rehner for the estimation of its different physico-chemical parameters that can lead us to gain insights about its network topology and how it behaves by changing the chain lengths of crosslinkers. These hydrogels are subsequently employed for evaluating model biological (BSA-TMR) release with high molecular weight model molecule and model dye (DY-781, lower molecular weight model molecule) release behavior, with an emphasis on the post-release dynamics of these delivered molecules for the first time.

## 2. Results and Discussion

### 2.1. Polymers and PSA-g-mPEG Hydrogel Syntheses

Linear polyester of poly(sorbitol adipate) was successfully synthesized through a polycondensation process driven by enzymatic catalysis (CAL-B) between sorbitol and divinyl adipate [24]. The linearity of the polymer is attained due to the highly reactive nature of the enzymes that react specifically and selectively with the primary functional groups rather than secondary functional groups [41]. This regioselective nature of enzymes enables the formed polymer to be utilized later on for crucial modifications. In this case, it leaves secondary hydroxyl groups to be used for further modifications. The regioselectivity of these enzymatically catalyzed reactions is attained by conducting polymer synthesis at lower temperatures rather than at higher temperatures. Synthesizing a polymer at a lower temperature leads to a linear polymer, while polymer synthesis at a higher temperature results in a branched polymer as well as a polymer with high molar mass [40,42]. Sorbitol was used in this polymer synthesis due to the fact that two of the primary hydroxyl groups are consumed during enzymatic polymerization, but its four secondary hydroxyl groups remain free for further modulation. These secondary hydroxyl groups were thus utilized for the modification of PSA by grafting with poly(ethylene glycol) (PEG) to enhance its water solubility [43]. PEG was selected due to the reason that it is a biocompatible polymer with better safety and a tunable profile. It is also widely used in the pharmaceutical industry for various purposes, e.g., drug delivery systems, solubility enhancers, stabilizers, etc. [44]. One of the many important purposes for its use inside the pharmaceutical industry is to graft it to biodegradable polymers and enhance the half-life of various drugs [43,44]. In addition to its benefits, PEG has been documented to trigger antibody formation within the human body, which may diminish the therapeutic effectiveness of the drugs [45,46].

### 2.2. Stability and Degradation Study of PSA and PSA-g-mPEG

Enzymatically based aliphatic polyesters have been reported as biodegradable, which means they break down to their initial monomeric products after degradation [47,48]. Studies suggest that post-polymerization modification can lead to a decrease in polymer degradation, which may be a result of an increase in the steric hindrance of the polymer [49]. We have also conducted a similar kind of study, in which polymers were exposed to two different types of temperatures to check their stability and degradation before (PSA) and after modification (PSA-*g*-mPEG).

**Figure 1:**
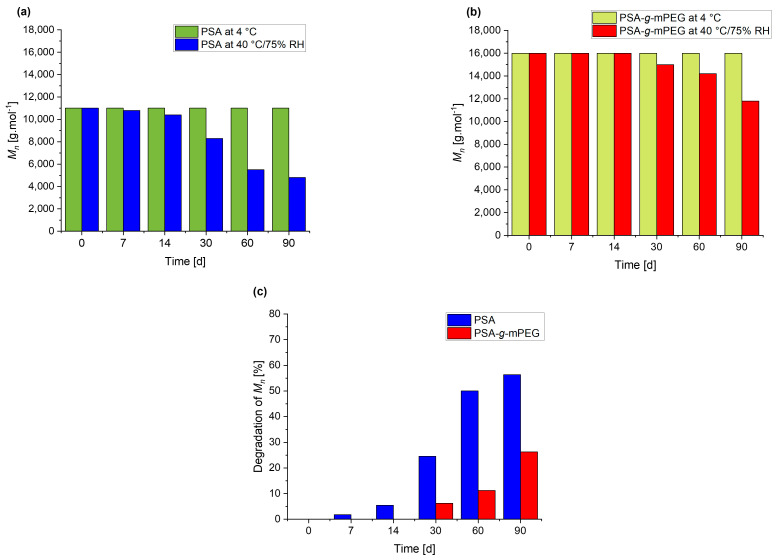
(**a**) Average molar mass (*M_n_*) of PSA at 4 °C and 40 °C/75% RH (Relative Humidity) with respect to time (84 d); (**b**) *M_n_* of PSA-*g*-mPEG at 40 °C/75% RH with respect to time (84 d); (**c**) % degradation of *M_n_* for PSA and PSA-*g*-mPEG at 40 °C/75% RH with respect to time (84 d).

The stability study suggests, through gel permeation chromatography (GPC) measurements, that no change was observed in the average molar mass (*M_n_*) before and after modifications when polymers were kept at 4 °C. In contrast to 4 °C, a decrease in molar mass was observed when these polymers were kept at 40 °C and 75% RH. As we can see in Figure 1a, the PSA initial molar mass was 11,000 g·mol^−1^ on day 0, but with the passage of time, it gradually decreased. It was reduced to 8400 g·mol^−1^ on day 28, while on day 84, it degraded to 4800 g·mol^−1^. A similar trend was observed with the PSA-*g*-mPEG (Figure 1b). On day 0, the molar mass of PSA-*g*-mPEG was 16,000 g·mol^−1^, which reduced slightly to 15,000 g·mol^−1^ on day 30, while it decreased to 11,800 g·mol^−1^ on day 84.

In both polymers (PSA and PSA-*g*-mPEG), the degradation of the polymer was observed at high temperature (40 °C) as compared to lower temperature (4 °C), which happens to be the result of hydrolysis [50,51,52]. The hydrolysis of ester bonds can be the result of high temperatures and humid conditions provided to the polymers. A degradation of the poly(glycerol adipate) (PGA), which is also a sugar alcohol-based polyester, has also been reported when it was exposed to similar environmental conditions [50]. It is also pertinent to mention here that PSA degrades more as compared to PSA-*g*-mPEG. If we compare the degradation of PSA and PSA-*g*-mPEG at day 84 (Figure 1c), PSA degraded to 43% of its initial molar mass, while PSA-*g*-mPEG degraded to 73% of its initial molar mass. This also justifies the fact that the modification of the polymer increases the steric hindrance of the cleavable ester bonds present in our polymer, which delays the degradation of the PSA-*g*-mPEG as compared to PSA [50,51,52]. A similar trend was also reported by Swainson et al. when they exposed poly(glycerol adipate) (PGA) and poly(glycerol adipate) modified with PEG (PGA-PEG) to enzymatic degradation. They found that PGA-PEG was more stable regarding the degradation effect as compared to PGA only, hence PEG providing the increase in the steric hindrance of the polymer [48]. Another potential scenario involves PEG acting as a polymer that can adhere to water due to its hygroscopic properties [53], thereby potentially reducing the amount of water accessible for hydrolysis and delaying the degradation process.

### 2.3. Sol-Gel Fraction of PSA-g-mPEG Hydrogels

**Figure 2:**
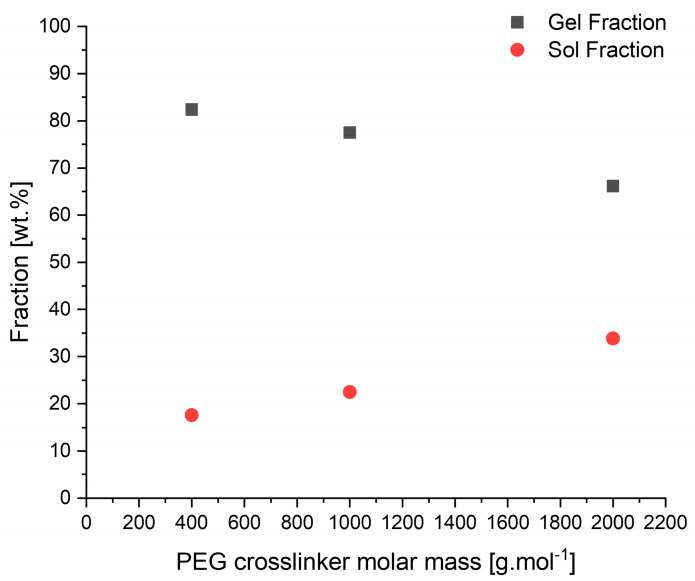
Sol-gel fraction in wt.% of hydrogels crosslinked with varying molar mass of PEG crosslinker.

**Scheme 1:**
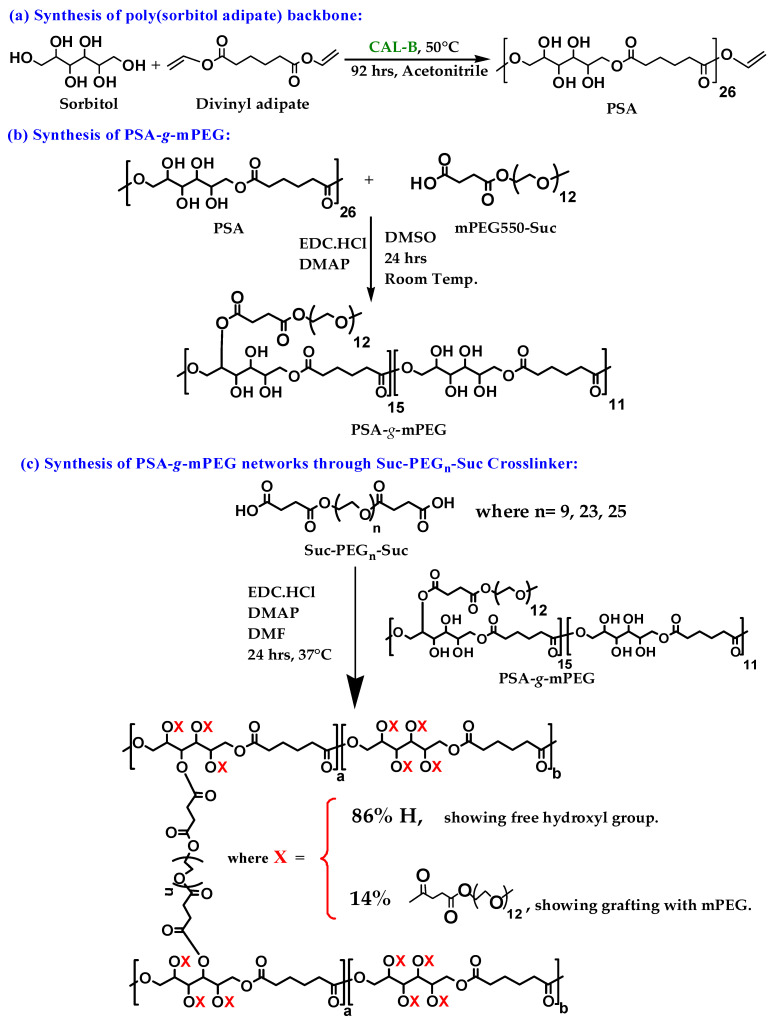
Synthesis scheme for hydrogel formation from PSA-*g*-mPEG and Suc-PEG_n_-Suc.

PSA-*g*-mPEG was then crosslinked through bifunctional PEG to form hydrogels using the Steglich esterification reaction. Three different types of crosslinkers were synthesized by using different chain lengths of PEG, i.e., PEG-400, PEG-1000, and PEG-2000. Crosslinker synthesis was achieved by esterifying hydroxyl groups of PEG and replacing them with carboxyl groups on both sides of PEG, making it a bifunctional crosslinking agent (Scheme 1). First of all, hydrogels were analyzed to identify crosslinked as well as uncrosslinked polymers/reactants during the reaction. This property can also tell us about the efficiency of the Steglich esterification reaction when it is used to form a crosslinked polymer network. So, to assess the amount of reactants consumed during the hydrogel formation, the sol-gel fraction of all the hydrogels was calculated. It can be revealed from Figure 2 that the gel percentage attained for the hydrogels crosslinked with PEG-400 was 82%; hydrogels crosslinked with PEG-1000 attained 77%, while the hydrogels crosslinked with PEG-2000 attained 66%. The sol% of all the mentioned hydrogels was attained as vice versa. According to Chen et al., an increase in crosslinking precursors can lead to an increase in grafting sites of the polymer network and cross-link density, which will eventually end up in a high-gel fraction [54]. In our case, the gel percentage is related to the chain length of the PEG. As the chain length of PEG-based crosslinkers is increased from PEG-400 to PEG-2000, the gel percentage is decreased. We can also explain our results in a way in which Steglich esterification proves to be more efficient when a lower chain length of PEG as a crosslinker is used as compared to the higher chain length of PEG. It is assumed that the probability of effective collision in a chemical reaction is decreased with the increase in chain length of PEG due to the high molar mass of PEG having a low enough degree of freedom to be mobile enough and react with the other chemical species [55].

### 2.4. Swelling Studies of PSA-g-mPEG Hydrogels

Swelling is an important phenomenon of hydrogels which renders them soft and elastic in nature, similar to natural tissues [56]. After coming into contact with the thermodynamically compatible solvent, the transition occurs from a glassy or partially rubbery state to a relaxed rubbery state. Hence, swelling can be described as a property of the polymer network that evolves due to the elasticity of the polymer chains, resulting from the interaction with a thermodynamically compatible solvent [57].

**Figure 3:**
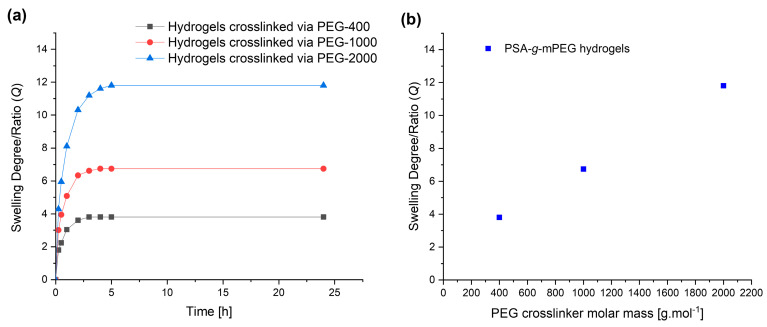
Hydrogel samples showing (**a**) dynamic swelling behavior and (**b**) equilibrium swelling behavior.

Due to this unique property, hydrogels have been studied in different biomedical applications [58]. The current experiment has been conducted to show the dynamic swelling as well as equilibrium swelling of our hydrogels. The swelling index demonstrates that the swelling of the hydrogels was dependent upon the varying chain length of the PEG-based crosslinkers (Figure 3). PEG-dependent swelling ratios have also been reported by various authors, illustrating that an increase in the molecular weight of the PEG leads to an increase in the molecular weight of the macromer. They further outline that an increase in the swelling of the system can not only be attributed to the enhancement in the overall hydrophilicity of the hydrogel system, but also to a decrease in crosslinking density [59,60,61]. In our case, the maximum swelling degree was observed in the hydrogels with a PEG-2000-based crosslinker, while the least swelling happened in the hydrogels with a PEG-400-based crosslinker. Equilibrium swelling for hydrogel samples crosslinked with all types of crosslinking agents was achieved within 4 h of the study.

### 2.5. Temperature-Dependent Swelling Behavior of PSA-g-mPEG Hydrogels

**Figure 4:**
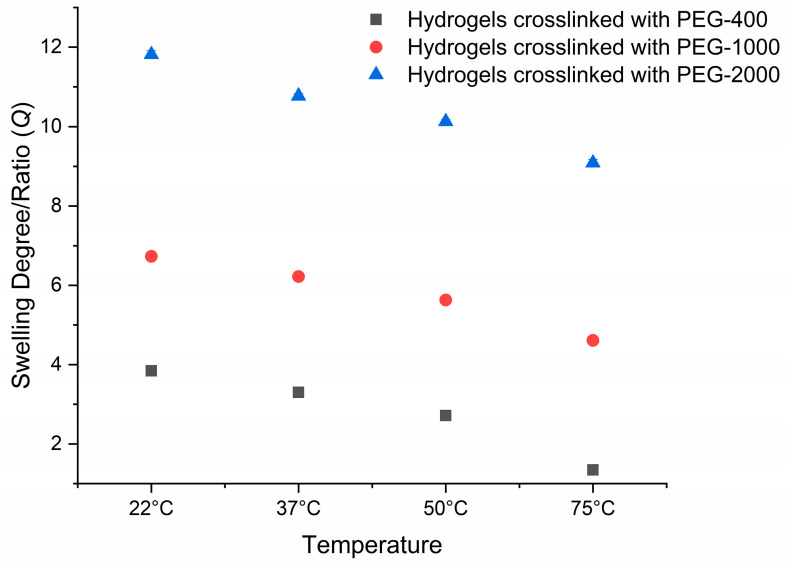
Swelling ratios of hydrogels as a function of temperature when it is increased from 22 °C to 75 °C.

The swelling of hydrogels was also investigated at different temperatures (22 °C, 37 °C, 50 °C, and 75 °C) to understand the effect of an increase in temperature over its swelling capability. It was revealed that the swelling ratio decreases with the increase in temperature [62,63,64]. Figure 4 shows that the swelling ratio, for all types of hydrogels crosslinked with different molar masses of PEG-based crosslinkers, decreased as the temperature was increased from 22 °C to 75 °C. The swelling ratio of hydrogels crosslinked with PEG-400 shrank from 3.84 at 22 °C to 1.32 at 75 °C, hydrogels crosslinked with PEG-1000 shrank from 6.73 at 22 °C to 4.60 at 75 °C, and hydrogels crosslinked with PEG-2000 shrank from 11.81 at 22 °C to 9.08 at 75 °C.

Such a behavior type has been explained as a PEG-based property [63,64,65]. Literature data further reveal that hydrogen bonding plays an important role in performing that behavior by forming hydrates when water comes into contact with PEG-based hydrogels. It is thus obvious that temperature would have a significant impact on the hydrogen bond formation between the PEG’s oxygen atom and the water’s hydrogen atom. Hence, such bonding is susceptible to breaking as the temperature rises, while interactions between hydrophobic molecules are strengthened [64,66,67,68].

### 2.6. Physical Structural Parameters of PSA-g-mPEG Hydrogels

The performance of hydrogels in a given application and their convenience as biomaterials relies heavily on their structural parameters. The most important of these parameters are *ʋ*_2*,s*_, ${\bar{M}}_{c}$, and *ξ*. *ʋ*_2,*s*_ relates to the polymer volume fraction of the hydrogels in a swollen state, which illustrates the capability of the hydrogel system to absorb solvent. It is determined by calculating the volume ratio of the dry polymer gel to the swollen gel. This property is also related to the reciprocal of the swollen gel ratio, and thus can be connected to the density of dry polymer gel, the density of the solvent, and the ratio of the swollen gel mass, as described in Equation (3).

${\bar{M}}_{c}$ outlines the degree of crosslinking of the hydrogel structure by estimating the molecular weight of the polymeric chain between two adjacent crosslinking points. It also gives us an idea about the crosslinking density of the hydrogel and how will it behave after interactions with the thermodynamically compatible solvent [69].

Macromolecular chains achieve their optimum configuration in the solvated state when they are cross-linked and swollen in a thermodynamically compatible solvent. This can be characterized by a correlation length, which measures how far apart crosslinks are on average from one another and indicates how the network affects solute diffusion. This distance is also referred to as mesh size, in which length is correlated to the diffusion of the solute. It can be challenging to experimentally determine this parameter and may require methods like light scattering [70,71] or in-depth microscopic examinations [72]. It can, however, be correlated to the theoretical estimation of molecular weight between two crosslinks (${\bar{M}}_{c}$) and polymer volume fraction (*ʋ*_2,*s*_). With the help of mesh size estimation, hydrogels can be classified as non-porous, microporous, and macroporous. This classification can also be used to understand and define the phenomena of degradation, solute diffusion, and mechanical toughness of the respective hydrogels. Various factors like crosslinking degree, monomer chemistry, and stimuli including pH, temperature, etc., can affect the mesh size of the hydrogel [73,74].

**Figure 5:**
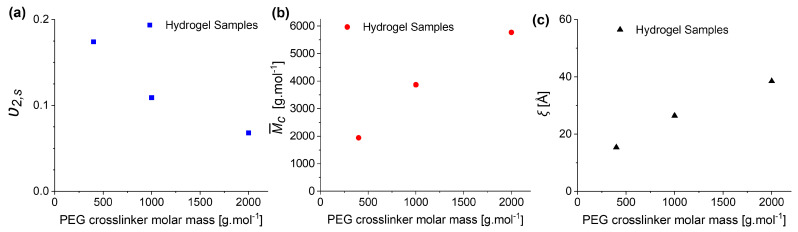
Graphs showing how (**a**) polymer volume fraction, (**b**) molecular weight between two crosslinks, and (**c**) mesh size of PSA-*g*-mPEG hydrogels varies by varying the chain length of PEG crosslinking agents.

The elucidation of these interrelated hydrogel structural parameters can be made theoretically with the help of the equilibrium swelling theory [75] and rubber elasticity theory [76]. It is worth mentioning here that these values can only be reported as average values due to the randomness of the polymerization process. We have calculated and explained these values with the help of the equilibrium swelling theory. It can be deducted from Table 1 and Figure 5a that as the swelling ratio (*Q*) increases with the increase in PEG-based crosslinker chain length, the polymer volume fraction (*ʋ*_2,*s*_) decreases, justifying the fact that polymer volume fraction (*ʋ*_2,*s*_) is inversely proportional to the swelling degree (*Q*) [71]. It was observed and reported by Waters et al. that using PEG macromonomers with higher molecular weight result in hydrogels with lower polymer volume fraction. This is due to the fact that PEG macromonomers with higher molecular weight lead to the formation of hydrogel networks with fewer cross-linking points per unit volume, resulting in weaker and less durable hydrogels [77].

The molecular weight between two crosslinks (${\bar{M}}_{c}$) of the hydrogels increased as the chain length of the PEG-based crosslinker increased, as shown in Figure 5b. This increase in the average molecular weight between two crosslinks of the hydrogels can be attributed to the chain length of the crosslinker, which increases from PEG-400 to PEG-2000. A similar trend in results was also reported by Troung et al. when they synthesized various chain lengths (from 1000 g·mol^−1^ to 20,000 g·mol^−1^) of PEG-based hydrogels through click chemistry [59].

The swelling ratio of a crosslinked polymer network demonstrates the thermodynamic expansion of the network, and the Flory–Rehner equation links this expansion to the network’s particular characteristics and structure [78]. Taking advantage of these findings, Canal and Peppas discovered that the average distance between crosslinks (expressed in angstroms), known as mesh size (*ξ*), can be determined using ${\bar{M}}_{c}$ [71]. It can be concluded from Figure 5c that the chain length of the crosslinkers utilized in the system is again a crucial factor in influencing the mesh size of our hydrogel system. Figure 5c shows that the hydrogel’s mesh size grows as the PEG-based crosslinker’s chain lengths are increased. These calculated values of mesh size or correlation length can also be somehow correlated to the length of the PEG-based spacers. As evident from previous studies, one unit of a short chain of PEG can be correlated to the length of 1.5 Å under a fully stretched helical structure [79]. Taking these calculations into account, if we look into the length of our PEG spacers, they make up 14 Å, 34 Å, and 67 Å for PEG-400, PEG-1000, and PEG-2000, respectively, under a fully stretched helical structure. We can thus deduce that the calculated lengths of 15 Å, 27 Å, and 38 Å of PEG-spacers can be correlated to the theoretical values of PEG-400, PEG-1000, and PEG-2000, respectively. As discussed earlier, these are the estimated values and are calculated on the assumption of a Flory-derived model. Here, measured values for the PEG-400 and PEG-1000 spacer correlate well with the theoretically calculated values, but the correlation length value for the PEG-2000 deviates a bit from the theoretical calculated value. Therefore, one can say that there is still room for improvement and the establishment of a more sophisticated model for the calculation of mesh size and other physical parameters of crosslinked polymeric networks in the case of copolymer-derived networks. On the other hand, these varying chain lengths of PEG-based spacers provide us with a basis for the modulation of mesh size as it varies, and thus can help in the diffusion of molecules from polymer networks. We can also comprehend the evidence that the relationship between the crosslinker’s chain length and mesh size can prove to be a vital factor in creating hydrogel systems with particular features to meet unique needs and requirements.

### 2.7. X-ray Diffraction

**Figure 6:**
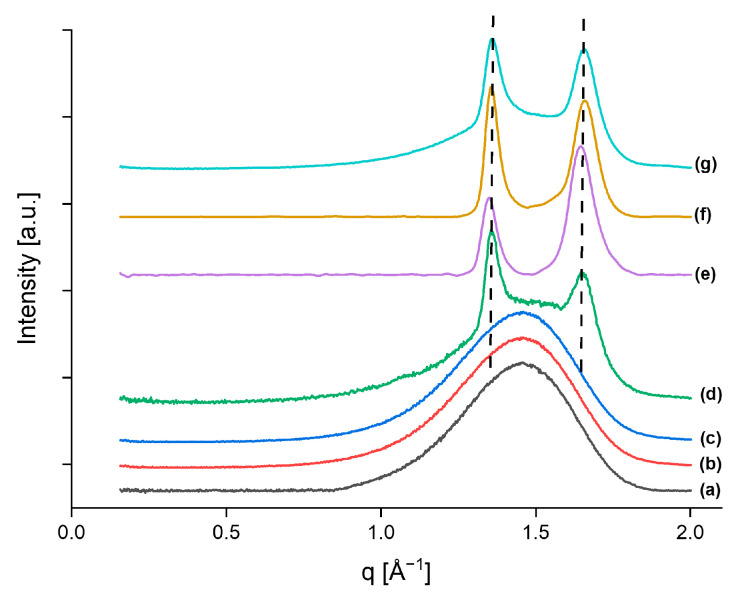
X-ray diffraction traces of (a) PSA-*g*-mPEG, (b) disuccinyl PEG-400, (c) dried PSA-*g*-mPEG hydrogels crosslinked with PEG 400, (d) disuccinyl PEG-1000, (e) dried PSA-*g*-mPEG hydrogels crosslinked with PEG-1000, (f) disuccinyl PEG-2000, and (g) dried PSA-*g*-mPEG hydrogels crosslinked with PEG-2000.

To discover the amorphous and crystalline characteristics of the hydrogel samples as well as the reactants responsible for the material’s crystallinity, X-ray diffraction measurements were performed on the samples. We can deduct from Figure 6 and Figure S8 that the neat polymer backbone (PSA-*g*-mPEG) and PEG-400-based crosslinker are completely amorphous in the spectra, while samples with PEG-1000-based and PEG-2000-based crosslinkers show significant crystalline peaks. The same reflections were also observed for neat PEG-1000 and PEG-2000. These correspond to the (120) and (032) Miller planes of monoclinic PEG, which are represented by the two characteristic peaks at *q* = 1.36 Å^−1^ and *q* = 1.66 Å^−1^, respectively. The unit cell is made up of PEG chains arranged in a 7_2_-helix structure, with seven repeating units in two turns [80]. Thus, we can conclude from the reactants/products data, as well as previous works in the literature, that the crystalline nature of our hydrogel samples is being induced by the PEG-based crosslinkers. These results are consistent with our previous findings investigated through differential scanning calorimetry results published elsewhere [24].

### 2.8. Loading and Release Experiment of BSA-TMR and DY-781 from PSA-g-mPEG Hydrogels

**Figure 7:**
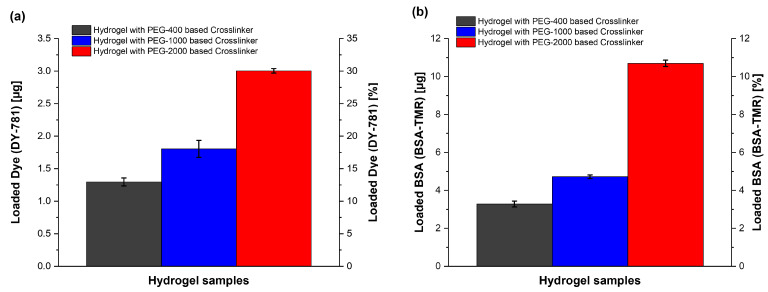
Post-loading efficiency of hydrogels through diffusion of molecules: (**a**) dye (DY-781) loading (µg) and (%), (**b**) BSA (BSA-TMR) loading (µg) and (%).

**Figure 8:**
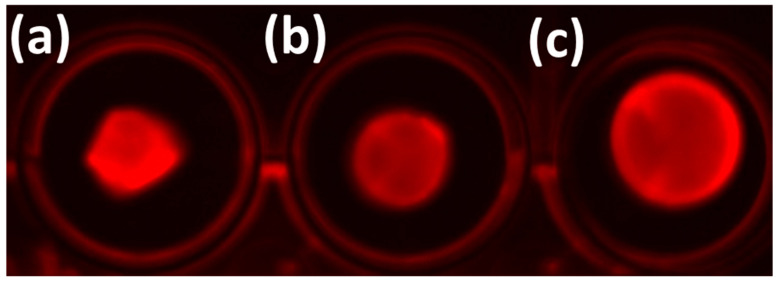
An illustration of how hydrogels look when dye is loaded inside. Post-loading fluorescence image of DY-784 into hydrogel matrices crosslinked with (**a**) PEG-400-based crosslinker, (**b**) PEG-1000-based crosslinker, and (**c**) PEG-2000-based crosslinker.

The loading of different molecules inside the hydrogels can be achieved by two different procedures. The first one is post-loading, which involves the movement of drug/protein/dye molecules from the outside (solvent solution) to the inside of already formed hydrogels, with diffusion being the major driving force. The second loading procedure is named in situ loading, and involves the mixing of drug/protein/dye molecules along with hydrogel precursors before its formation [69,81,82]. We adopted the post-loading method, so that clean hydrogels without unwanted polymer traces can be used. To assess the loading and release of lower and higher molecular weight molecules from hydrogel matrices, we used two different model molecules. The lower molecular weight molecule used was DY-781, a fluorescent dye with a molecular weight of 781 g·mol^−1^, while the higher molecular weight molecule used was BSA-TMR (a model protein, bovine serum albumin conjugated with a dye, i.e., tetramethyl-rhodamine), with a molecular weight of 66,000 g·mol^−1^. Loaded BSA-TMR and DY-781 were analyzed using the fluorescence spectrometer. In both DY-781 and BSA-TMR, loading was directly related to the swelling of the hydrogel samples. Hydrogels with the maximum swelling (PSA-*g*-mPEG crosslinked with PEG-2000-based crosslinker) attained the highest loading, while the hydrogel sample with the lowest swelling index (PSA-*g*-mPEG crosslinked with PEG-400-based crosslinker) achieved the lowest loading (Figure 7 for loading and Figure 8 for swelling). Thus, the loading of protein/dye molecules was directly related to the varying molar mass of the PEG crosslinker. Furthermore, the theoretically estimated mesh size, as shown in Figure 5c, also gives an idea that the diffusion of molecules through the hydrogels can be correlated to the mesh size of the chain length of the PEG-based crosslinker. Considering the estimated mesh size of the hydrogels and the loaded quantity of molecules as delineated in Figure 7, it is inferred that small-sized molecules are being loaded to a greater extent than large-sized molecules. This may be attributable to the fact that the mesh size of the hydrogels is larger than the small-sized molecules, whereas in the case of BSA, the mesh size of the hydrogels somehow matches the hydrodynamic radii of BSA: 34 Å [61].

**Figure 9:**
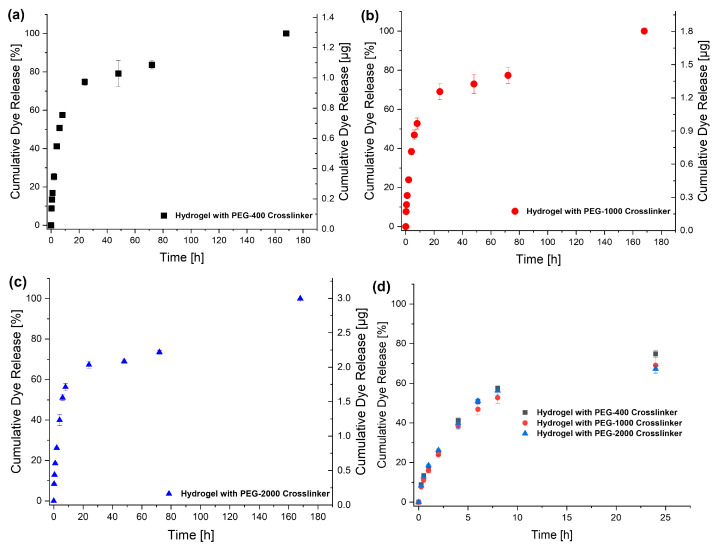
DY-781 cumulative release (%) in PBS with pH 7.4 + 0.2% NaN_3_ at 37 °C: (**a**) from hydrogels with PEG-400 crosslinker for 168 h (07 d), (**b**) from hydrogels with PEG-1000 crosslinker for 168 h (07 d), (**c**) from hydrogels with PEG-2000 crosslinker for 168 h (07 d), and (**d**) from all hydrogel samples for 24 h.

**Figure 10:**
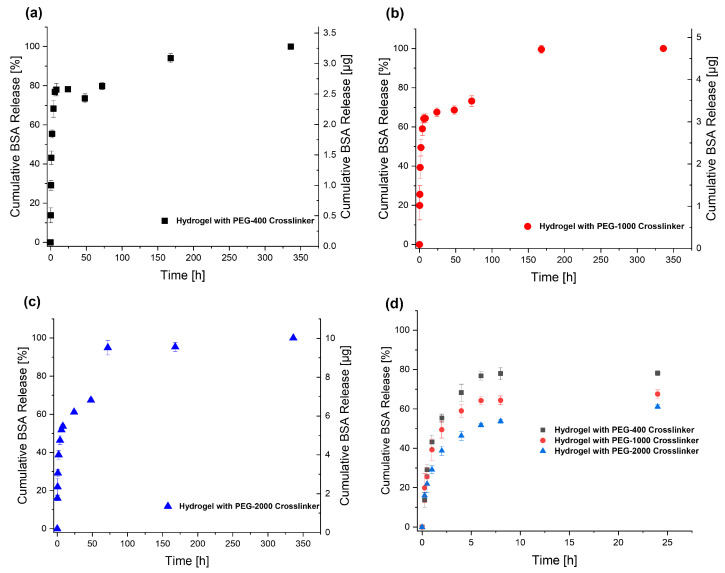
BSA (BSA-TMR) cumulative release (%) in PBS with pH 7.4 + 0.2% NaN_3_ at 37 °C: (**a**) from hydrogels crosslinked with PEG-400 for 336 h (14 d), (**b**) from hydrogels crosslinked with PEG-1000 for 336 h (14 d), (**c**) from hydrogels crosslinked with PEG-2000 for 336 h (14 d), and (**d**) initial release period from all hydrogel samples for 24 h.

The in vitro release study of DY-781 (Figure 9) and BSA-TMR (Figure 10) from hydrogels was performed at 37 °C in PBS pH 7.4. Before the release study of BSA-TMR, a fluorescence investigation of a blank hydrogel sample (degraded/solution form) and hydrogel sample (degraded/solution form) along with BSA-TMR was carried out to understand the interaction between hydrogel and BSA-TMR. The fluorescence spectrum was recorded within the range of tetramethyl rhodamine (TMR), with an excitation at 535 nm and an emission at 576 nm. The fluorescence spectrum (Supplementary Materials Figure S9) clearly shows that there was no interaction recorded between hydrogel and BSA-TMR and the spectrum only shows the TMR peak within the applied fluorescence range. Release data of DY-781 demonstrate (Figure 9) that 40% of the DY-781 was released from all hydrogel matrices after 4 h of release study, which can be attributed to the equilibrium swelling of hydrogel samples. The hydrogel swelling index (Figure 3) can be correlated to its release data as maximum swelling (equilibrium swelling) of the hydrogels occurred after 4 h. Witnessing the same pattern as for swelling, it can be seen that the largest amount of DY-781 was released from the hydrogel matrices in the same period of time. The majority of DY-781 was released after 24 h of study (Figure 9d), in which hydrogels crosslinked with PEG-400 showed a 75% release and hydrogels crosslinked with PEG-1000 showed 69%, whereas hydrogels crosslinked with PEG-2000 showed 67% release. A 100% DY-781 release was recorded after one week of the study.

The release study of BSA-TMR shows an almost similar pattern of release to that of DY-781, in that its release from hydrogel matrices continued for almost 14 days, and showed a 100% release on the 14th day (Figure 10). Again, the highest percentage of release can be attributed to the swelling pattern of the hydrogels. After 4 h of release study, hydrogels with a PEG-400-based crosslinker released 68% BSA-TMR and PEG-1000-based crosslinked hydrogels released 59% BSA-TMR, while PEG-2000-based crosslinked hydrogels released 46% BSA-TMR. After 24 h of BSA-TMR release (Figure 10d), hydrogels crosslinked with PEG-400 demonstrated 78% release and hydrogels with a PEG-1000-based crosslinker demonstrated 67% release, whereas hydrogels crosslinked with PEG-2000 showed 61% release. The release of BSA-TMR continued for 14 days.

BSA-TMR and DY-781 release from different types of hydrogels crosslinked with the different chain lengths of PEG crosslinkers cannot be compared with each other as there was a difference in the loaded amount in both cases. In addition, if we look into BSA-TMR and DY-781 release individually, there was a difference in the loaded amount of the protein/dye molecules for all hydrogels crosslinked with varying molar mass of PEG. We can still deduce that molecular release from these hydrogel matrices is connected to the swelling of the hydrogels that occurs due to the interaction between water molecules and polymer chains. This interaction firstly leads to the diffusion of the water molecules inside the polymer hydrogels, which initially loosens up the polymer chains and ends up in the expansion of the hydrogel systems due to the relaxation of the polymer chains [83,84], leading to the increase in the mesh size and desorption of the model molecule. Our current data show that swelling plays a major part in molecular release, as most of the molecular release is achieved within 24 h. We further assume that this swelling-controlled release mechanism may simultaneously be followed by a chemically controlled mechanism due to the fact that our polymer precursors consist of vinyl end groups which may interact with the amines present in the protein/dye molecules. This interaction can lead to Aza–Michael addition under mild reaction conditions without the presence of any catalyst, which was also reported by Razan et al. [85]. Thus, some of the protein/dye molecular release may be attributed to the chemically controlled release mechanism in which molecules may release after hydrolytic degradation of the hydrogels as they swell after coming into contact with the water. Such a type of release mechanism can be termed a reaction diffusion-controlled mechanism in which both diffusion and chemical reaction take place [69]. For this reason, the fast release of molecules through the hydrogel pores was first experienced, followed by slow release later on. Adding to this assumption, we previously conducted proton double quantum NMR studies on our hydrogel system, which proves that our PSA-*g*-mPEG hydrogel stands inhomogeneously due to the grafting of PEG side chains that can act as dangling chains. These NMR studies also tell us about the two components present in our hydrogels; one is densely crosslinked regions with more crosslinking junctions, while the second is loosely crosslinked regions with lesser crosslinking junctions. This may lead to the trapping of molecules in these densely crosslinked points, which may take some time to diffuse outside [24]. Considering the pharmaceutical perspective, the release patterns observed with BSA-TMR and DY-781 offer promising prospects for the potential dermal and oral applications of these hydrogel matrices. These applications may involve the need for immediate drug release during the initial phase, ensuring an immediate therapeutic effect followed by a delayed release in the later stages of drug delivery, maintaining a consistent therapeutic concentration over time and prolonging the efficacy of the treatment.

### 2.9. Cytotoxicity of PSA-g-mPEG Hydrogels

Material biocompatibility is vital for in vivo applications [86], while cytotoxicity can provide important insights about material biocompatibility [87]. To investigate the cytotoxicity of our hydrogels (degraded solution form), an in vitro resazurin assay was conducted in which the reduction of non-fluorescent blue resazurin to fluorescent red resorufin within living cellular mitochondria was measured. The comparative analysis of fluorescence intensity between treated and untreated cells enabled an assessment of cellular viability after its interaction with our hydrogels. This assay was carried out on two different cell lines, normal human dermal fibroblasts (NHDF) and mouse embryonic fibroblasts (3T3), to measure the cytotoxic traits of our hydrogels without loaded protein/dye.

**Figure 11:**
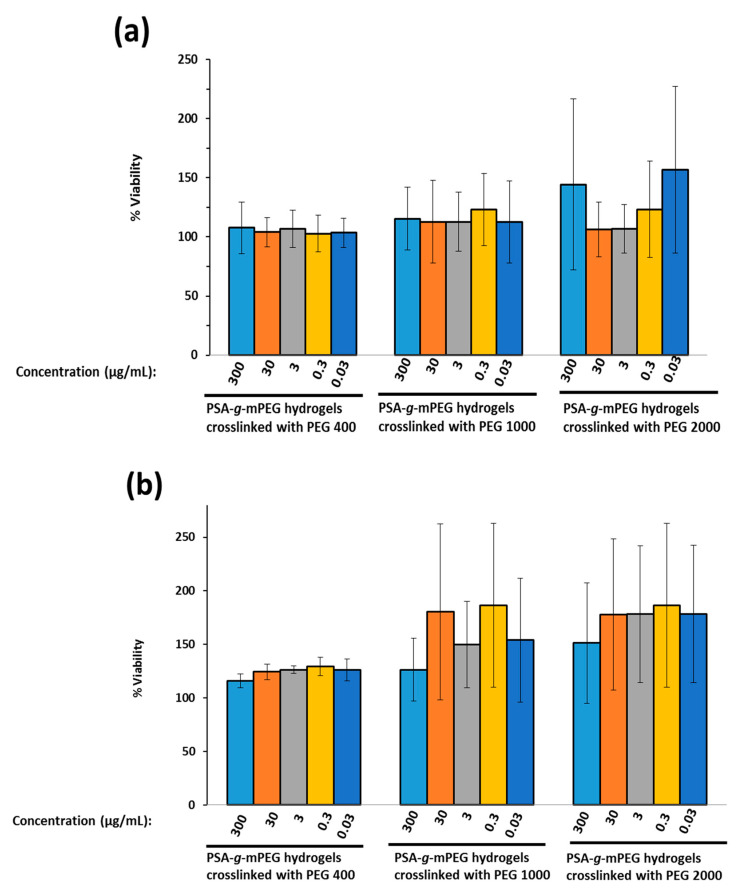
Viability of 3T3 cell line after being incubated with different concentrations of different types of hydrogels for (**a**) 4 h and (**b**) 96 h. The measurement was performed using a fluorescence-based resazurin reduction assay and the viability of untreated cells was taken as the reference (negative control) and set as 100%.

**Figure 12:**
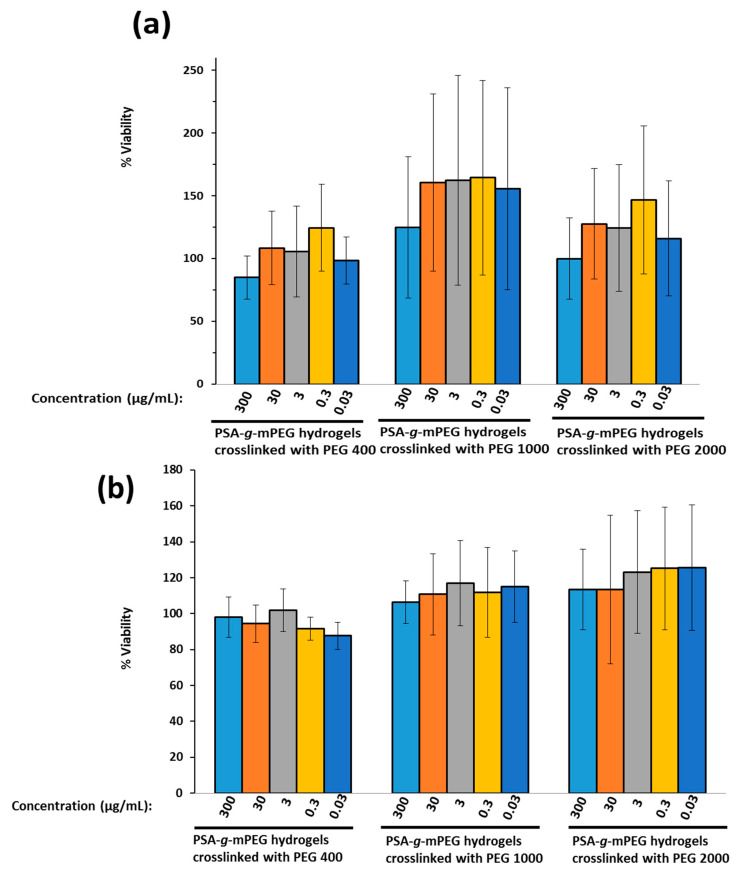
Viability of NHDF cell line after being incubated with different concentrations of different types of hydrogels for (**a**) 4 h and (**b**) 96 h. The measurement was performed using a fluorescence-based resazurin reduction assay and the viability of untreated cells was taken as the reference (negative control) and set as 100%.

Figure 11 and Figure 12 depict percentage cell viabilities for the 3T3 cell line and NHDF cell line, respectively, of different hydrogel samples at various concentrations after its incubation for 4 h and 96 h. We can evaluate from the results that both types of cell lines exhibit a high percentage of cell viability for all hydrogel samples against different concentrations. It can also be indicated that a cell viability of above 100% was observed across most of the hydrogel samples after 96 h. This can be interpreted as our hydrogel samples indicating the enhancement of cell metabolism and proliferation. Also, few of the prior research studies indicate that sorbitol has been reported as a factor that might play an important role in cell metabolism and growth in in vitro cell compatibility studies. Mei et al. conducted the same type of study by culturing 3T3 cell lines with polyesters. After 24 h of cell culture study, they analyzed sorbitol-containing polyesters and found that they can show cell proliferation, concluding that sorbitol-containing compounds can prove to be promising candidates in the research and development of biomaterials [88]. In another investigation, conducted on human skin fibroblasts, Turner and Biermann came to the conclusion that sorbitol can proliferate similarly to glucose [89]. Keeping in view our obtained results of cytotoxicity as well as previous results from the literature, we may deduce that our product may prove to be biocompatible in conducting in vivo experiments and for the development of biomaterials.

## 3. Conclusions

Enzymatic polymerization was employed for the synthesis of green aliphatic polyesters with various advantages. Sorbitol was used instead of glycerol (having been used by our research group previously) because of its multi-hydroxyl pendant functionalities. These multiple pendant functionalities make sorbitol more versatile to be modulated later for multiple purposes. Utilizing this advantage, it was first copolymerized with PEG to make it more water-soluble, followed by crosslinking to synthesize a hydrophilic polymer network. Both PSA and PSA-*g*-mPEG hydrogels were first investigated for stability studies and it was found that PSA-*g*-mPEG degrades slowly as compared to PSA, due to the steric hindrance provided by PEG to the PSA backbone. The swelling properties of PSA-*g*-mPEG hydrogels revealed that the swelling ratio is directly proportional to the chain length of the PEG-based crosslinkers from PEG-400 to PEG-2000. Temperature-based swelling properties revealed that the swelling ratio of the hydrogels decreases due to the breakage of hydrogen bonding as the temperature rises from RT to 75 °C. The sol-gel fraction of the hydrogels tells us that the crosslinking efficiency of the reaction is inversely proportional to the chain length of the PEG-based crosslinkers. The determination of the physical structural parameters explains that they are also dependent upon the chain length of the PEG-based crosslinkers. Solute-loading studies confirm that the hydrogel with the most swelling ratio has the highest efficiency in taking up solute, with up to 30% of dye and 10% of BSA-TMR with PEG-2000 through the diffusion mechanism. Solute release from the hydrogels was fast during the initial phase, while it became delayed in the later phase. Cytotoxicity studies revealed the biocompatibility of the polymers and thus can be exploited for in vivo studies against measured concentrations. We can thus assume that our hydrogel system can prove to be a potential candidate for various biomedical and pharmaceutical applications while considering the versatile nature of our polymer, its physicochemical and solute release properties, and biocompatibility.

## 4. Materials and Methods

### 4.1. Materials

CAL-B (Novozyme 435), which is a lipase derived from Candida Antarctica type B and immobilized on acrylic resin, was purchased from Sigma Aldrich, St. Louis, MO, USA. Prior to its use, it was first vacuum-dried over phosphorous pentoxide for 24 h. Sorbitol (98%) and divinyl adipate (96%) were purchased from Sigma Aldrich (Steinheim, Germany) and TCI GmbH (Eschborn, Germany), respectively. Phosphorous pentoxide (≥99%), 4-(dimethylamino)pyridine (DMAP), anhydrous *N*,*N*-dimethylformamide (DMF, 99.8%), anhydrous tetrahydrofuran (THF, 99.9%), acetonitrile (anhydrous, 99.8%), 1-ethyl-3-(3-dimethylaminopropyl) carbodiimide hydrochloride (EDC·HCl), dialysis membranes with 1000 g·mol^−1^ molecular weight cut off (MWCO), and 10,000 g·mol^−1^ MWCO (Spectra/Por^®^, made from regenerated cellulose) were purchased from Carl Roth, Karlsruhe, Germany. α,ω-bis-hydroxy poly(ethylene glycol)_n_ (where n = 9, 23, and 45) and α- methoxy,ω-hydroxy poly(ethylene glycol)_n_ (where n = 12) (mPEG 550) were purchased from Alfa Aesar, Kandel, Germany. DY-781 Amine (molecular weight: 781 g·mol^−1^) and DY-784 NHS-ester (molecular weight: 1188 g·mol^−1^) were purchased from Dyomics GmbH (Jena, Germany), while BSA-TMR was purchased from Thermo Fisher Scientific Inc. (Waltham, MA, USA).

### 4.2. Polymers and Hydrogel Syntheses

Prior to the formation of polymer networks, poly(sorbitol adipate) was synthesized via enzymatic polymerization, as described earlier in Rashid et al., 2021 [24]. Briefly, 50 mL of anhydrous acetonitrile was added to a three-necked round bottom flask with the equimolar amount of sorbitol (10.0 g, 54.9 mmol) and divinyl adipate (10.88 g, 54.9 mmol), whose central neck was connected to a mechanical stirrer while one of the side necks was connected to a reflux condenser with a calcium chloride drying tube. The round bottomed flask was placed in an oil bath. The purpose of the drying tube was to absorb atmospheric moisture while allowing the venting of acetaldehyde, a side product of this reaction. The solution was stirred for half an hour so that the temperature of the reaction flask was equilibrated to 50 °C. Lipase [Novozyme 435, 2.1 g (10% *w*/*w* of total mass of PSA and DVA)] was then added to the solution, which initiated the reaction. After 92 h, the reaction was stopped and the mixture diluted with DMF. Enzyme beads were removed through filtration with Whatmann^®^ filter paper. The filtrate was then transferred to a dialysis membrane of 1K MWCO and dialysis of the polymer was performed with water as the dialysis medium. Water was exchanged thrice per day for 7 days to remove oligomers of the product. The final product was achieved through freeze drying of the dialyzed polymer. The product’s purity was verified using ^13^C NMR (Figure S2) and ^1^H NMR spectroscopy (Supplementary Materials Figure S1a). ^1^H NMR (400 MHz, DMSO-d_6_) δ (ppm): 4.95–4.58 (m, 2H), 4.57–4.33 (m, 2H), 4.28–3.86 (m, 2H), 3.82–3.72 (m, 2H), 3.61–3.34 (m, 2H), 2.38–2.18 (m, 4H), and 1.61–1.41 (m, 4H).

To achieve a higher swellability of the hydrogels, PSA was grafted to poly(ethylene glycol) (PEG). For the grafting of PSA to PEG, mPEG 550 was modified by acylation with succinic anhydride via a procedure described elsewhere [24,90] to obtain monofunctional PEG (Supplementary Materials Figure S4). ^1^H-NMR ((400 MHz, CDCl_3_) δ (ppm): 4.25–4.21 (m, 2H), 3.68–3.51 (m, 50H), 3.35 (s, 3H), 2.67–2.56 (m, 4H)). PSA was then grafted to PEG through the Steglich esterification reaction mentioned elsewhere [24], where monosuccinyl methoxy PEG reacted with PSA to produce PSA-*g*-mPEG. It is important to mention here that the polymer batch used for the stability study was different from the batch used for the synthesis of hydrogels. The average molar mass (*M_n_*) of PSA used in the stability study was 11,000 g·mol^−1^, while the *M_n_* of PSA used for the preparation of hydrogels was 7500 g·mol^−1^. Similarly, the *M_n_* of PSA-*g*-mPEG utilized for the stability study was 16,000 g·mol^−1^, while the *M_n_* of PSA-*g*-mPEG utilized for the preparation of hydrogels was 17,250 g·mol^−1^ (Supplementary Materials Figures S3 and S1b (^13^C NMR) (^1^H NMR)). ^1^H NMR ((400 MHz, DMSO-d_6_) δ (ppm) (Figure S1b)): 4.95–4.58 (m, 2H), 4.57–4.33 (m, 2H), 4.16–4.08 (m, 2H), 4.28–3.86 (m, 3H), 3.82–3.72 (m, 2H), 3.56–3.45 (m, 50H), 3.23 (s, 3H), 2.61–2.52 (m, 4H), 3.61–3.34 (m, 2H), 2.38–2.18 (m, 4H), 1.61–1.41 (m, 4H).

The next step was to crosslink the PSA-*g*-mPEG polymer through a bifunctional PEG, which took place by first synthesizing bifunctional PEG. The PEG of three different molar masses (PEG-400, PEG-1000, and PEG-2000) was acylated on both sides with succinic anhydride through a procedure described elsewhere [24,90,91]. In the last step, hydrogels were formed by esterifying secondary hydroxyl groups from PSA-*g*-mPEG and terminal carboxyl groups from bifunctional PEG (Supplementary Materials Figures S5–S7). ^1^H NMR ((400 MHz, CDCl_3_) δ (ppm): 4.28–4.20 (m, 4H), 3.73–3.57 ((m, 34H (bifunctional PEG 400); 92H (bifunctional PEG 1000); 180H (bifunctional PEG 2000)), 2.68–2.58 (m, 8H). Steglich esterification took place by using EDC⋅HCl and DMAP. In a typical procedure, PSA-*g*-mPEG (1.00 g, 13.7 mmol) was taken and dissolved at 37 °C in DMF (w.r.t 22% *w*/*v* of the combined weight of PSA-*g*-mPEG plus crosslinker). This was subsequently followed by the addition of DMAP (0.34 g, 2.72 mmol) and EDC⋅HCl (4.72 g, 27.38 mmol) in a vial. Three different bifunctional crosslinkers were added to three different solutions and were kept overnight at 37 °C without stirring. Then, 35 mol% of crosslinkers was added to the solution, which was calculated with respect to the free hydroxyl groups present on the PSA-*g*-mPEG backbone. Hydrogels were then formed and cut into cylindrical discs. The purification of the hydrogels was carried out by washing the gel discs in double-distilled water for 7 days while replacing the washing medium thrice per day. Impurities were washed out and swollen gel discs were dried in a drying oven at 37 °C. The synthesis scheme for the hydrogels is given below as Scheme 1.

### 4.3. Polymer Degradation/Stability Study

Polymers were exposed to 2 different types of temperatures. One part was placed at 4 °C in a fridge, while the second part was placed at 40 °C with a relative humidity (RH) of 75% to check its degradation and stability. For this purpose, 5 mg of the polymer was taken at each time point and samples were kept in the fridge and Heraeus B 6760 climate chamber (Thermo Fisher Scientific Inc., Waltham, MA, USA). At various time points (days), samples were taken and measured through gel permeation chromatography (GPC). For GPC measurement, samples were analyzed at room temperature by using Viscotek GPCmax VE 2002. Briefly, 5 mg/mL of sample was taken and dissolved in DMF (along with 0.01 M LiBr). For calibration of the instrument, poly(methyl methacrylate) was used while a 1 mL.min^−1^ flow rate of eluent was adopted for the measurement. Samples were finally analyzed by a determination of the average molar mass (*M_n_*).

### 4.4. Sol-Gel Fraction of PSA-g-mPEG Hydrogels

Sol-gel fraction investigation was performed to learn about the crosslinked and uncrosslinked portions of hydrogels. For that, hydrogel samples after synthesis containing uncrosslinked polymers, catalysts, and solvent were immersed in double-distilled water. Water was replaced 3 times per day for an interval of one week, to remove the uncrosslinked portions of the hydrogels. It is pertinent to mention here that all the reactants involved in the preparation of the hydrogels were soluble in water. Finally, hydrogels were dried and weighed. The sol-gel fraction of hydrogels was calculated with the help of the following equations, where *W*_0_ refers to the initial weight of hydrogel precursors before the reaction, while *W*_1_ refers to the final weight of dried clean hydrogels after washing [92,93].

(1) $$\mathrm{Gel}\;\mathrm{fraction}\;(\mathrm{wt}.\%)=\frac{W_{0}-W_{1}}{W_{0}}\times 100$$

Sol fraction (wt.%) = 100 − Gel fraction,(2)

### 4.5. Swelling Studies

The swelling study of the hydrogel matrices was initiated by immersing hydrogels in water. Hydrogels were first weighed in a dry state, followed by taking the weight of the swollen discs at regular time intervals for 24 h at room temperature (22 °C) until they reached their equilibrium swelling state. For weighing the hydrogels, they were taken out of the solvent every time. Surface water was removed by rolling over the blotting paper. Dynamic and equilibrium swelling degrees were calculated by taking into account the initial dry weight and swollen weights of hydrogels at different time intervals [59]. Moreover, equilibrium swelling degrees for all samples were also calculated at room temperature, 37 °C, 50 °C, and 75 °C to evaluate and investigate the effect of increasing temperature on our hydrogel as general physicochemical characteristics of this polymeric system.

The swelling degree was determined using Equation (3), where *m_t_* refers to the swollen hydrogels, while *m*_o_ refers to the dried hydrogel discs.

(3) $$Q=\frac{m_{t}-m_{o}}{m_{o}},$$

### 4.6. Structural Parameters of the PSA-g-mPEG Hydrogels

Swelling measurements were then utilized to calculate various physical parameters related to the polymeric structure of the hydrogel system. One of these important physical parameters is ${\bar{M}}_{c}$, the molecular weight between two crosslinks which allows for determining the degree of crosslinking between the polymeric chains. ${\bar{M}}_{c}$ can be calculated through modified Flory–Rehner’s theory by using Equation (4) [59,94].

(4) $$\frac{1}{{\bar{M}}_{c}}=\frac{2}{{\bar{M}}_{n}}-\frac{\frac{\nu_{1}}{V_{1}}\left[\ln\left(1-\nu_{2}\right)+\nu_{2,s}+\chi_{1}\nu_{2,s}^{2}\right]}{\left[{\left(\nu_{2,s}\right)}^{\frac{1}{3}}-\left(\frac{2}{\varphi }\right)\nu_{2,s}\right]}$$

Here, ${\bar{M}}_{n}$ represents the polymer’s average molecular weight prior to the crosslinking, i.e., for PSA-*g*-mPEG (17,500 g·mol^−1^), $\nu_{1}$ is the specific volume of the polymer, $V_{1}$ is the molar volume of the solvent, *φ* is the functionality of the crosslinker, i.e., 2, $\chi_{1}$ is the polymer-solvent interaction parameter also known as the Flory–Huggins parameter or chi parameter, and $\nu_{2}$ is the polymer volume fraction in the swollen state. To solve this equation, the polymer solvent interaction parameter ($\chi_{1}$) of PEG was taken, which was 0.426. We made this assumption based on the fact that the molecular weight of the PEG in our hydrogel system was larger compared to the PSA and the swelling properties of our hydrogel system were driven by PEG. A similar type of assumption has also been reported elsewhere [61]. The specific volume of the polymer, $\nu_{1}$, was determined by calculating the density of the polymer hydrogels. The molar volume of the solvent ($V_{1}$) is 18.1 mL/mol. The polymer volume fraction tells us about the efficiency of our hydrogel systems to absorb water. It is calculated by taking the volume ratio of the dry hydrogels to the swollen hydrogels, which can be related to the degree of swelling as well as to the densities of the hydrogels and solvent. It can be calculated [59] by using the following Equation (5).

(5) $$\nu_{2,s}=\frac{\rho_{s}}{Q\rho_{p}+\rho_{s}}$$

Here, $\rho_{s}$ is the density of the solvent, while $\rho_{p}$ is the density of the dry hydrogel and Q is the degree of swelling.

Once ${\bar{M}}_{c}$ is calculated, it is easy to determine the mesh size or correlation length of the respective hydrogels. The correlation length (*ξ*) is a common structural parameter used to describe the size of the pores of the hydrogels. It represents the linear distance between two neighboring crosslinks and can be calculated [69,73] through the value of

(6) $$\xi ={v_{2,s}}^{-1/3}{\left(\bar{{r_{0}}^{2}}\right)}^{1/2}$$

where *υ*_2,*s*_ is the polymer volume fraction of the hydrogels, while $\bar{{r_{0}}^{2}}$ is the end-to-end distance between two adjacent crosslinking points and can be determined as

(7) $${\left(\bar{{r_{0}}^{2}}\right)}^{1/2}=l{\left(C_{n}N\right)}^{1/2}$$

Here, *Ɩ* is the bond length, which was assumed to be the average bond length of one PSA repeating unit, i.e., 1.51 Å. *C_n_* is the Flory characteristics ratio that tells us about the flexibility or rigidity of the polymer chain [78]. Since the Flory characteristics ratio of PSA has not been determined to date, we are assuming here the *C_n_* of a polyamide, Nylon 6,6 (6.1) [95], whose one repeating unit has almost the same length as of PSA, while *N*, the number of links per chain, can be determined as

(8) $$N=\frac{2{\bar{M}}_{c}}{M_{r}}$$

where ${\bar{M}}_{c}$ refers to the molar mass between the two crosslinks, while *M_r_* is the molar mass of one repeating unit of the polymer chain. Here, we assumed the molar mass of one repeating unit of PSA-*g*-mPEG, i.e., 640 g·mol^−1^.

### 4.7. X-ray Diffraction (XRD) of PSA-g-mPEG Hydrogels

Wide-angle X-ray scattering (WAXS) measurements were performed with an Incoatec IµS (Geesthacht, Germany) equipped with a microfocus source and a monochromator for Cu K_α_ radiation (λ = 0.154 nm). The 2D scattering patterns were recorded using a Vantec 500 2D detector (Bruker, AXS, Karlsruhe, Germany). The samples were kept in glass capillaries of 1 mm diameter (manufactured by Hilgenberg, GmbH, Malsfeld, Germany), while network samples were measured in transmission mode with 1 mm thickness. The exposure time was 3 min. The distance between the sample and detector was 9.85 cm for wide-angle scattering experiments.

### 4.8. Loading Study of the BSA-TMR and DY-781 into Hydrogel Matrices

In order to assess the release patterns of lower and higher molecular weight molecules from hydrogel matrices, DY-781 as a lower molecular weight molecule (molecular weight: 781 g·mol^−1^) and model protein bovine serum albumin conjugated with tetramethyl rhodamine (BSA-TMR) (molecular weight: 66,000 g·mol^−1^) as a high molecular weight molecule were loaded into hydrogels as follows. In detail, BSA-TMR solution was prepared by dissolving 100 μg of BSA-TMR in 1 mL phosphate-buffered saline with pH 7.4 at a concentration of 0.1 mg/mL (PBS), while DY-781 was prepared by dissolving 10 μg of DY-781 in 1 mL phosphate-buffered saline (PBS) with pH 7.4 at a concentration of 0.01 mg/mL. Different dried hydrogel samples with a diameter of 3 mm (PSA-*g*-mPEG crosslinked with disuccinyl PEG-400, PSA-*g*-mPEG crosslinked with disuccinyl PEG-1000, and PSA-*g*-mPEG crosslinked with disuccinyl PEG-2000) were then placed in these solutions for 48 h. Hydrogels were allowed to swell so that BSA-TMR and DY-781 could diffuse inside hydrogel samples. After 48 h, hydrogels were then subjected to freeze drying in order to obtain dried hydrogel samples. The loading efficiency was calculated, keeping in view the concentration of the initially prepared solutions and loaded concentration inside hydrogels measured through fluorescence spectroscopy by using a FluoroMax-4 spectrofluorometer (Details Section 4.9). Additionally, DY-784 was also loaded through the same method as described before for an illustration of dye-loaded hydrogels. The Maestro™ imaging system (Cambridge Research & Instrumentation Inc., Hopkinton, MA, USA) was used to capture fluorescence images by using a near-infrared filter set. A filter set designed for near-infrared (NIR) wavelengths, including a 710 nm to 760 nm excitation filter and an 800 nm long-pass emission filter, was employed to capture the DY-784 signal. Image cubes were systematically acquired in 10 nm increments spanning the range of 780 to 950 nm. The analysis of these images was conducted using Maestro™ software (Version 2.10.0). The exposure time was automatically optimized, and the software correlated the total fluorescence signal to the corresponding value.

### 4.9. Release Study of the BSA-TMR and DY-781

BSA-TMR- and DY-781 -loaded hydrogel samples were then subjected to a release study in order to evaluate their release from these hydrogel matrices. For this purpose, dried hydrogel samples were taken and placed in glass vials with conserved PBS pH 7.4 as the release medium. Glass vials were placed in a shaking water bath at 60 rpm and 37 °C temperature. The water bath was protected from the sunlight. Then, 500 μL of aliquots were taken at different time intervals and were replaced with the same volume of fresh PBS in order to maintain the sink conditions of the release media. BSA-TMR and DY-781 aliquots were then analyzed through fluorescence spectroscopy by using a FluoroMax-4 spectrofluorometer (HORIBA Jobin Yvon GmbH, Bensheim, Germany). The detection of the DY-781 signal involved a single-point acquisition with an excitation wavelength of 784 nm and an emission wavelength of 796 nm, while for BSA-TMR the excitation wavelength was 535 nm and the emission length was 576 nm. Measurements were conducted in a 10 mm quartz cuvette, and the data obtained were analyzed using FluorEssence™ software (HORIBA Jobin Yvon GmbH, Version 3.8.0.60). The final calculation of the release data was performed by constructing a calibration curve of BSA-TMR and DY-781. The experiment was performed in triplicate.

### 4.10. Cytotoxicity Study of PSA-g-mPEG Hydrogels

In vitro cell toxicity studies were performed for all three hydrogel samples: PSA-*g*-mPEG crosslinked with disuccinyl PEG-400, PSA-*g*-mPEG crosslinked with disuccinyl PEG-1000, and PSA-*g*-mPEG crosslinked with disuccinyl PEG-2000. Hydrogels were exposed to 37 °C for a longer period of time until they degraded to solution form in PBS. Two different cell lines were used. The first was the 3T3 cell line, which is a murine embryonic fibroblast cell line originally isolated from kidney tissue, while the second cell line used was NHDF, which is a normal human dermal fibroblast cell line originally isolated from a human foreskin sample.

Seeding of the cells took place in 96 well plates (TPP^®^ tissue culture test plate flat bottom, TPP Techno Plastic Products AG, Trasadingen, Switzerland). These cells were grown in an incubator (Heraeus HeraCell CO_2_ incubator, Thermo Fisher Scientific Inc., Waltham, MA, USA) in 100 μL culture media at 37 °C and 5% CO_2_ overnight. In the case of the NHDF cell line, the culture medium used for the cultivation of the cells consisted of Dulbecco’s Modified Eagle Medium (DMEM; Sigma-Aldrich GmbH, Taufkirchen, Germany), 10% (*v*/*v*) fetal bovine serum (FBS, Sigma-Aldrich GmbH, Taufkirchen, Germany) and 1% penicillin/streptomycin solution (Sigma-Aldrich GmbH, Taufkirchen, Germany). Then, 4 mM sodium pyruvate was added in addition for the culture medium in case of the 3T3 cell line. Column 1 of the well plate was left blank without any seeding of cells to obtain the background signal. Column 2 was considered as a negative control, as cells were left untreated to obtain 100% vitality, while column 3 was made the positive control by treating cells with 0.05% (*v*/*v*) Triton^®^ X-100 solution to obtain 0% vitality (or 100% cell death). In the remaining columns of the well plates, hydrogel solutions were added to the cells and incubated for 4 h, 24 h, and 96 h.

A Resazurin assay was used to determine the viability in % by the metabolic activity of the cells. Briefly, 20 µL of Resazurin solution (440 µM) was added to the well plate, followed by its incubation for 2 h. The well plate was then placed in a multi-mode cell imaging reader (Cytation™ 5 cell imaging reader, BioTek Instruments Inc., Winooski, VT, USA). Using a filter set with an excitation wavelength of 531 nm and an emission wavelength of 593 nm, fluorescence intensities were recorded. The final cell viability percentage was then determined by taking into account the negative control after subtracting the blank (background signal).

## Figures and Tables

**Table 1:** Degree of swelling (*Q*), polymer volume fraction (*ʋ*_2,*s*_), molecular weight between two crosslinks (${\bar{M}}_{c}$), and mesh size (*ξ*) of hydrogel samples.

Hydrogels	*Q*	*ʋ*_2__,*s*_	${\bar{M}}_{c}$(g·mol^−1^)	*ξ*(Å)
Hydrogels crosslinked with PEG-400	3.81	0.174	1941	15
Hydrogels crosslinked with PEG-1000	6.74	0.109	3869	26
Hydrogels crosslinked with PEG-2000	11.80	0.068	5772	38

## Data Availability

Data are contained within the article and Supplementary Materials.

## Supplementary Materials

The following supporting information can be downloaded at: https://www.mdpi.com/article/10.3390/gels10010017/s1, Figure S1: ^1^H NMR spectra of (a) PSA and (b) PSA-*g*-mPEG measured at 27 °C using DMSO-d_6_ as solvent. Figure S2: ^13^C NMR spectrum of poly(sorbitol adipate) measured at 27 °C using DMSO-d_6_ as solvent. Figure S3: ^13^C NMR spectrum of PSA-*g*-mPEG measured at 27 °C using DMSO-d_6_ as solvent. Scheme S1: Synthesis scheme of mPEG-Suc. Figure S4: ^1^H NMR spectrum of mPEG-Suc measured at 27 °C using CDCl_3_ as solvent. Scheme S2: Synthesis scheme of Suc-PEG_n_-Suc. Figure S5: ^1^H NMR spectrum of disuccinyl PEG-400 (Suc-PEG_9_-Suc) measured at 27 °C using CDCl_3_ as solvent. Figure S6: ^1^H NMR spectrum of disuccinyl PEG-1000 (Suc-PEG_23_-Suc) measured at 27 °C using CDCl_3_ as solvent. Figure S7: ^1^H NMR spectrum of disuccinyl PEG-2000 (Suc-PEG_45_-Suc) measured at 27 °C using CDCl_3_ as solvent. Figure S8: X-ray diffraction scattering patterns of (a) PSA-*g*-mPEG, (b) Disuccinyl PEG-400, (c) PSA-*g*-mPEG hydrogels crosslinked with PEG-400, (d) Disuccinyl PEG-1000, (e) PSA-*g*-mPEG hydrogels crosslinked with PEG-1000, (f) Disuccinyl PEG-2000, and (g) PSA-*g*-mPEG hydrogels crosslinked with PEG-2000. Figure S9: Fluorescence spectra of BSA-TMR along with hydrogel’s degraded sample and degraded hydrogel without BSA-TMR measured via fluorescence spectrometer within wavelength range of excitation (535 nm) and emission (576 nm) to evaluate interaction between both or any background signal.
