# Supplementary material for: Development of Poly(sorbitol adipate)-g-poly(ethylene glycol) Mono Methyl Ether-Based Hydrogel Matrices for Model Drug Release
Source: Gels. 2023 Dec 23;10(1):17. doi: 10.3390/gels10010017 (PMC10815636; doi:10.3390/gels10010017)
Supplement: Supplementary file 1 [file gels-10-00017-s001.zip › gels-2781499-supplementary.pdf]

## Supplementary Materials:

# Development of Poly(Sorbitol Adipate)-*g*-Poly(Ethylene Glycol) Monomethyl Ether based Hydrogel Matrices for Model Drug Release

**Haroon Rashid[1,2], Henrike Lucas[1], Karsten Busse[2], Jörg Kressler[2], Karsten Mäder[1] and Marie-Luise Trutschel[1,*]**

[1] Institute of Pharmacy, Martin Luther University Halle-Wittenberg, D-06120 Halle (Saale), Germany; haroon.rashid@student.uni-halle.de (H.R.); henrike.lucas@pharmazie.uni-halle.de (H.L.); karsten.maeder@pharmazie.uni-halle.de (K.M.)

2 Department of Chemistry, Martin Luther University Halle-Wittenberg, D-06120 Halle (Saale), Germany; karsten.busse@chemie.uni-halle.de (K.B.); joerg.kressler@chemie.uni-halle.de (J.K.)

* Correspondence: marie-luise.trutschel@pharmazie.uni-halle.de (M.-L.T.); Tel.: +49-345-552-5146

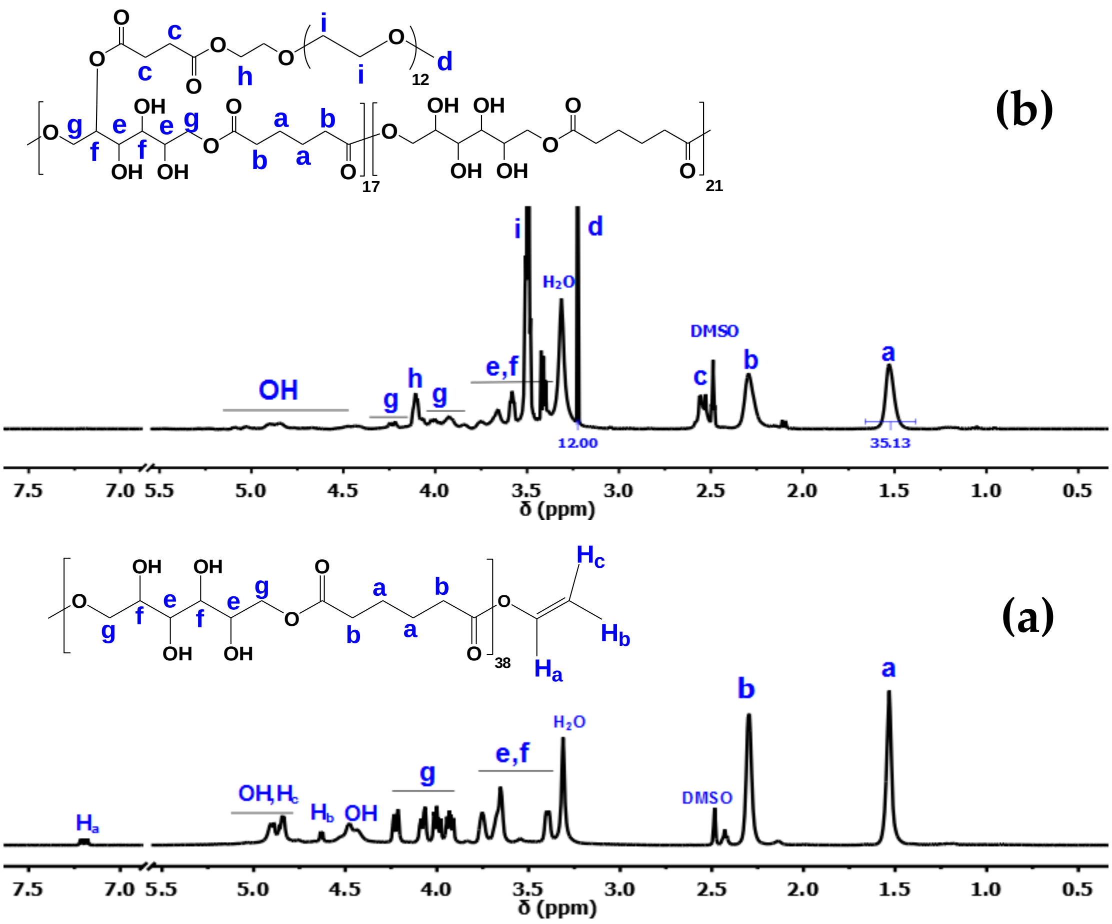


**Figure S1.** $^1$H NMR spectra of (a) PSA and (b) PSA-*g*-mPEG measured at 27 °C using DMSO-$d_6$ as solvent.

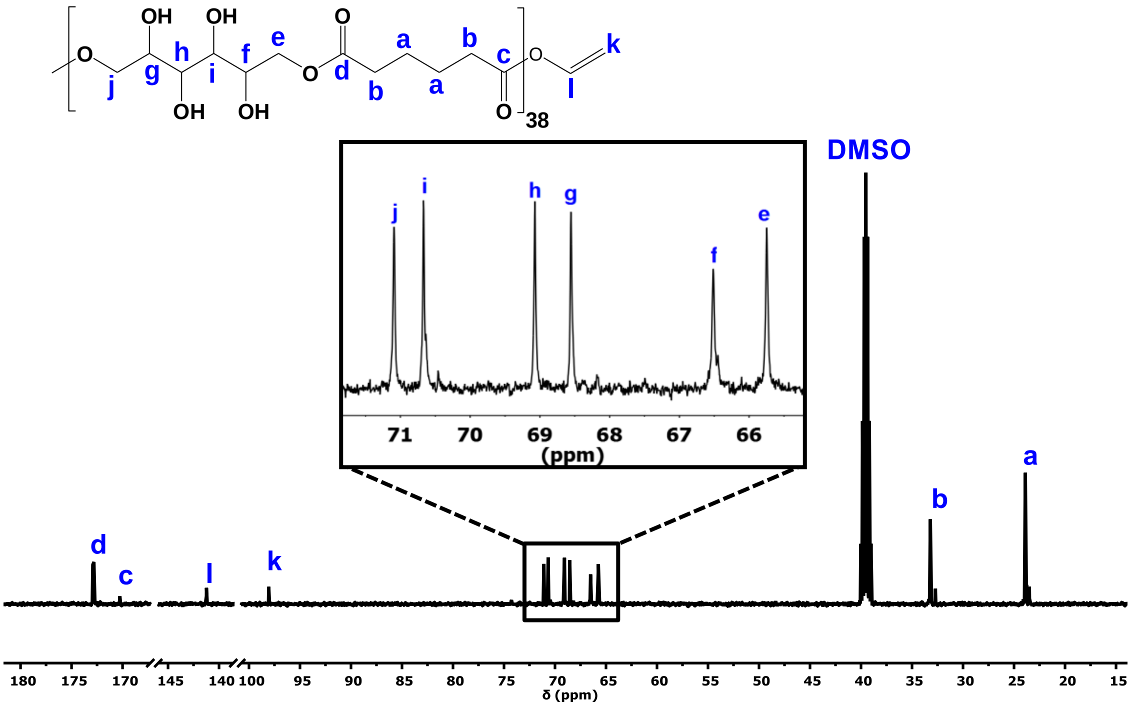


**Figure S2.** $^{13}$C NMR spectrum of poly(sorbitol adipate) measured at 27 °C using DMSO-d$_6$ as solvent.

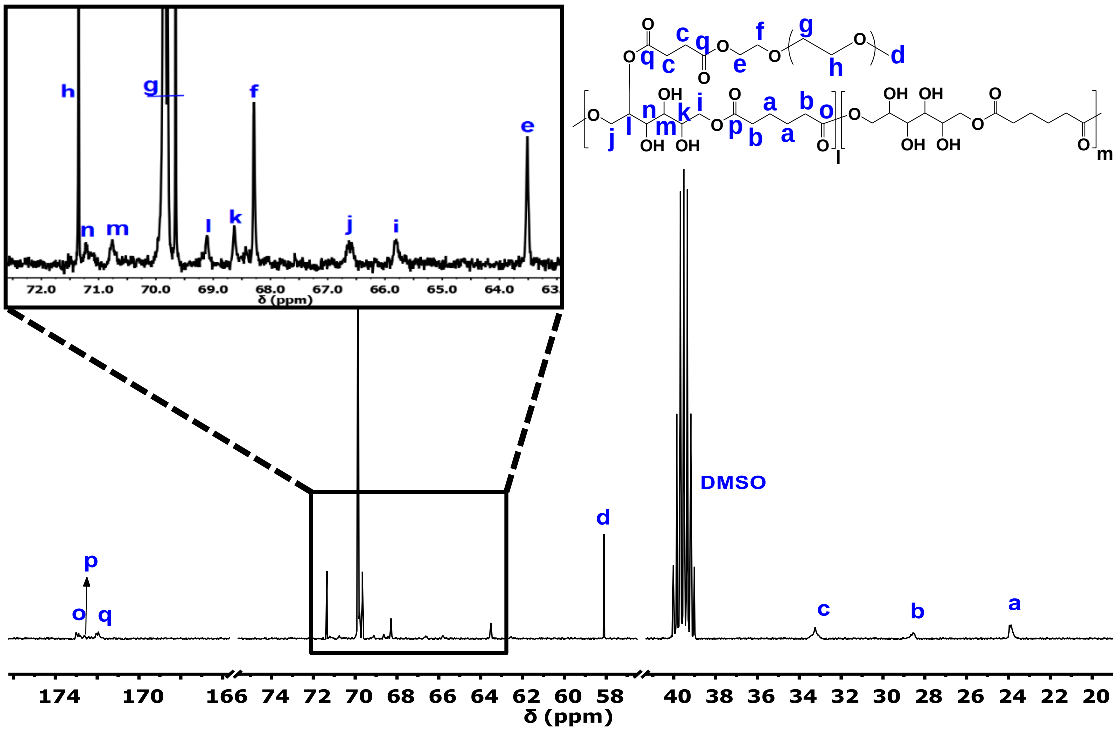


**Figure S3.** $^{13}$C NMR spectrum of PSA-*g*-mPEG measured at 27 °C using DMSO-d$_6$ as solvent.

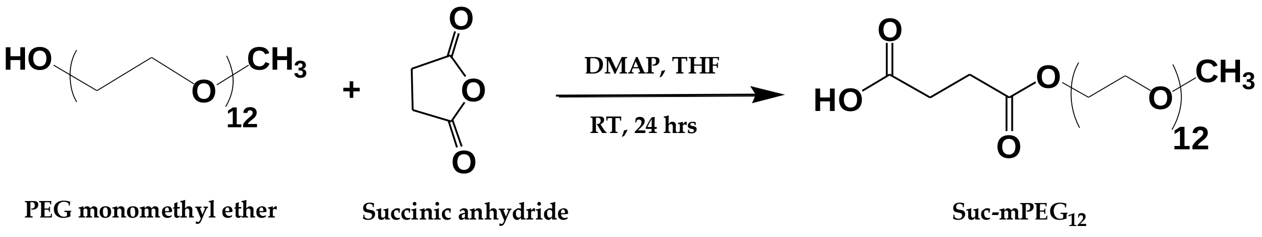


**Scheme S1.** Synthesis scheme of mPEG-Suc

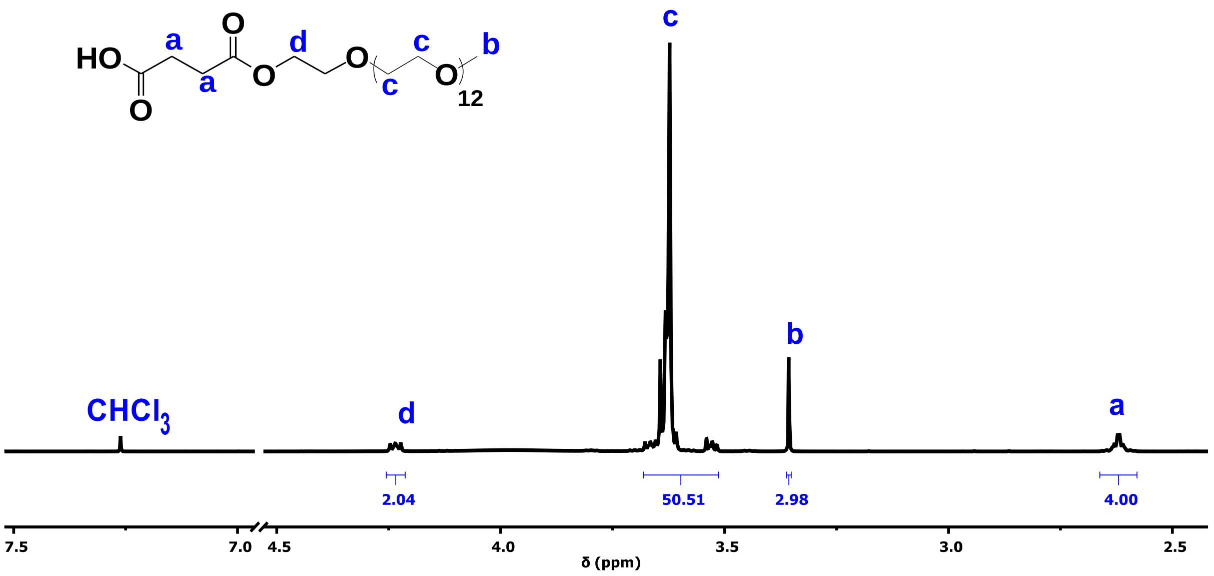


**Figure S4.** $^1$H NMR spectrum of mPEG-Suc measured at 27 °C using $CDCl_3$ as solvent.

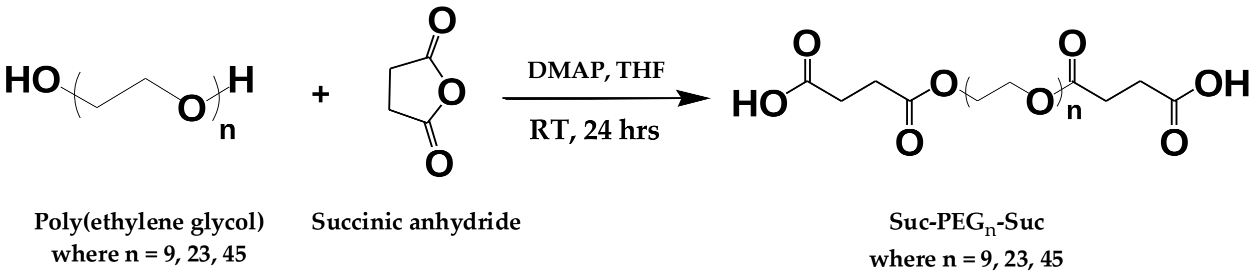


**Scheme S2.** Synthesis scheme of Suc-PEG$_n$-Suc.

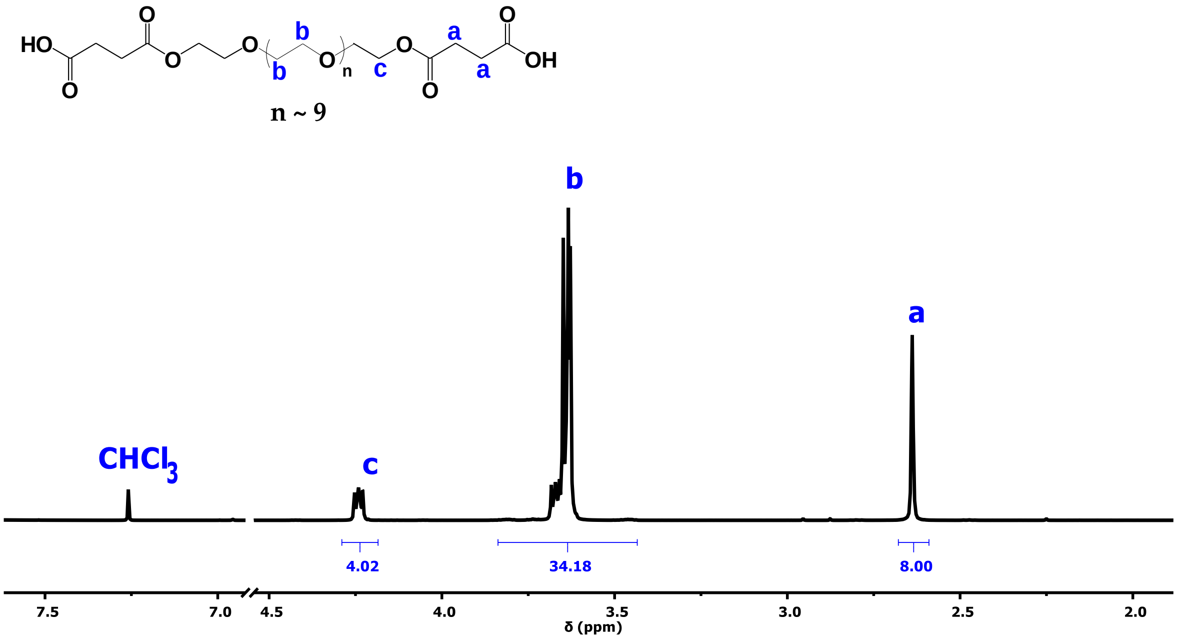


**Figure S5.** $^1$H NMR spectrum of disuccinyl PEG-400 (Suc-PEG$_9$-Suc) measured at 27 °C using $CDCl_3$ as solvent.

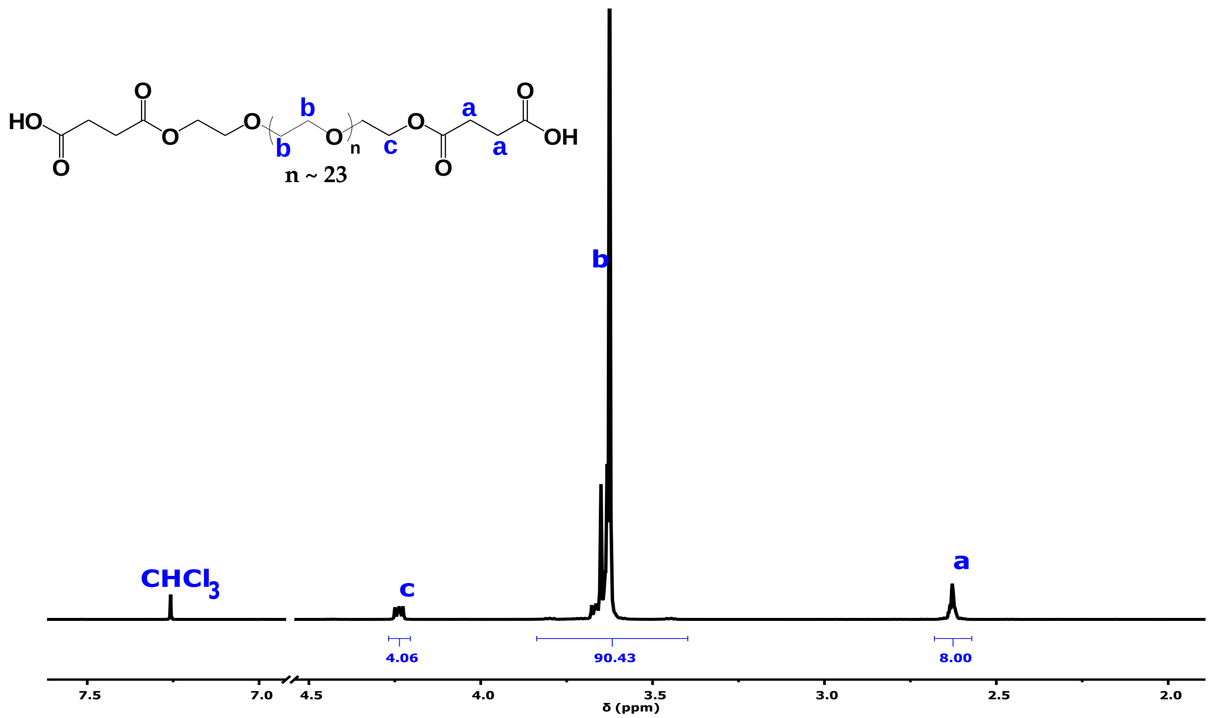


**Figure S6.** $^{1}$H NMR spectrum of disuccinyl PEG-1000 (Suc-PEG$_{23}$-Suc) measured at 27 °C using $CDCl_3$ as solvent.

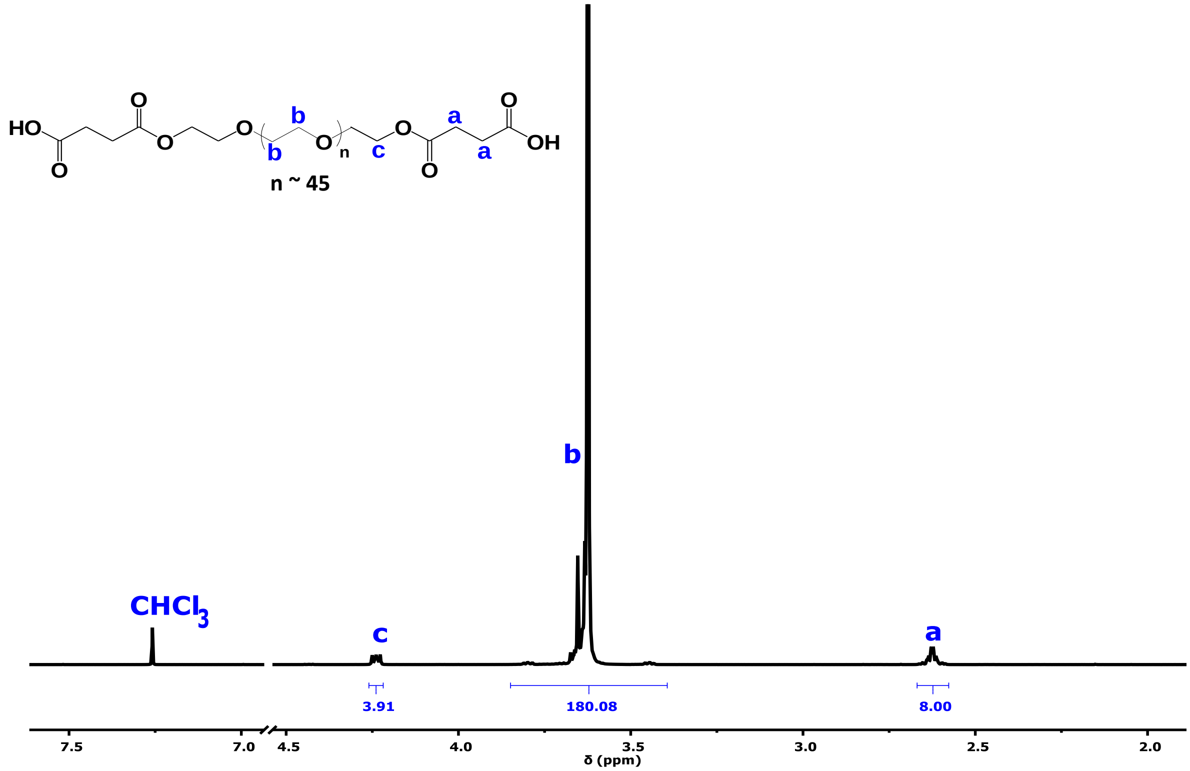


**Figure S7.** $^{1}$H NMR spectrum of disuccinyl PEG-2000 (Suc-PEG$_{45}$-Suc) measured at 27 °C using $CDCl_3$ as solvent.

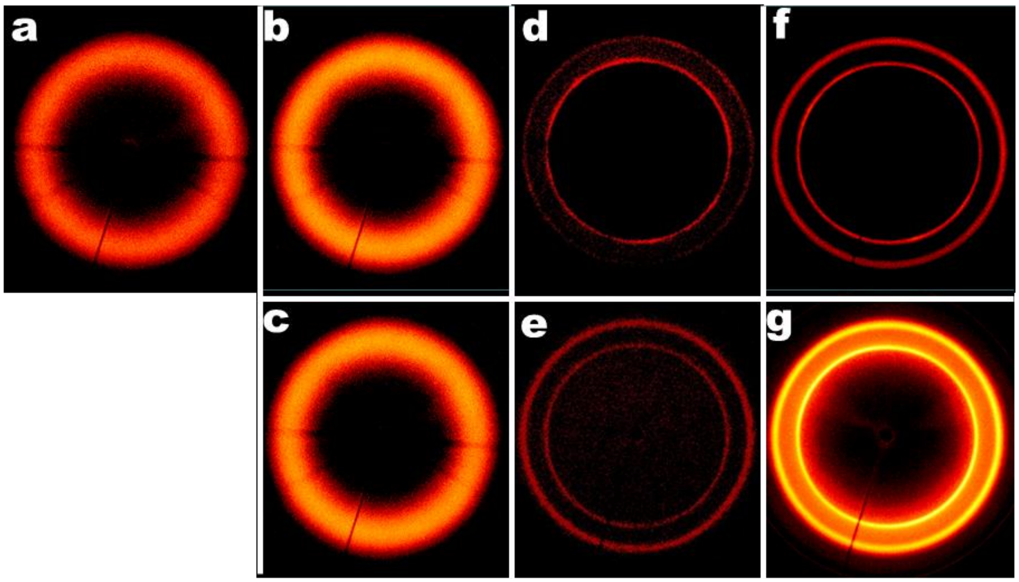


**Figure S8:** X-ray diffraction scattering patterns of (a) PSA-*g*-mPEG (b) Disuccinyl PEG-400 (c) PSA-*g*-mPEG hydrogels crosslinked with PEG-400 (d) Disuccinyl PEG-1000 (e) PSA-*g*-mPEG hydrogels crosslinked with PEG-1000 (f) Disuccinyl PEG-2000 (g) PSA-*g*-mPEG hydrogels crosslinked with PEG-2000.

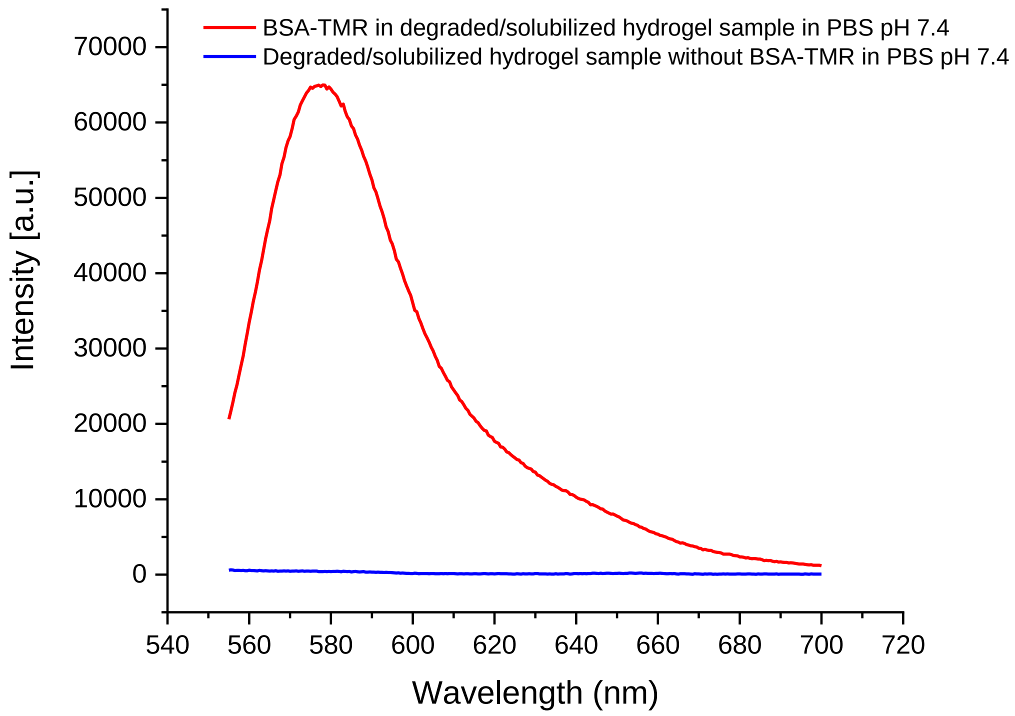


**Figure S9:** Fluorescence spectra of BSA-TMR along with hydrogel's degraded sample and degraded hydrogel without BSA-TMR measured via fluorescence spectrometer within wavelength range of excitation: 535 nm and emission: 576 nm, to evaluate interaction between both or any background signal.
